# Using an AI-based avatar for interviewer training at Children’s Advocacy Centers: Proof of Concept

**DOI:** 10.1177/10775595241263017

**Published:** 2024-06-18

**Authors:** Gunn-Astrid Baugerud, Miriam S. Johnson, Rachel Dianiska, Ragnhild K. Røed, Martine B. Powell, Michael E. Lamb, Syed Zohaib Hassan, Saaed S. Sabet, Steven Hicks, Pegah Salehi, Michael A. Riegler, Pål Halvorsen, Jodi Quas

**Affiliations:** 1 418298 Oslo Metropolitan University, Oslo, Norway; 2 8788 University of California Irvine, Irvine, CA, USA; 3 95796 Griffith University, Mount Gravatt, QLD, Australia; 4 2152 University of Cambridge, Cambridge, UK; 5 571514 Simula Metropolitan Center for Digital Engineering AS, Lysaker, Norway

**Keywords:** child advocacy Centers, child abuse, forensic interviews, interviewing children, sexual abuse, interview techniques

## Abstract

This proof-of- concept study focused on interviewers’ behaviors and perceptions when interacting with a dynamic AI child avatar alleging abuse. Professionals (*N* = 68) took part in a virtual reality (VR) study in which they questioned an avatar presented as a child victim of sexual or physical abuse. Of interest was how interviewers questioned the avatar, how productive the child avatar was in response, and how interviewers perceived the VR interaction. Findings suggested alignment between interviewers’ virtual questioning approaches and interviewers’ typical questioning behavior in real-world investigative interviews, with a diverse range of questions used to elicit disclosures from the child avatar. The avatar responded to most question types as children typically do, though more nuanced programming of the avatar’s productivity in response to complex question types is needed. Participants rated the avatar positively and felt comfortable with the VR experience. Results underscored the potential of AI-based interview training as a scalable, standardized alternative to traditional methods.

The burgeoning field of technology-enhanced learning has offered an array of solutions to traditional educational constraints in various disciplines, including psychology (Chernikova et al., 2020; Crompton et al., 2020). One example is the introduction and use of Artificial Intelligence (AI), which has yielded numerous benefits for students’ learning and had a major impact on the education sector (Chen et al., 2020). We have been exploring a novel application of AI, specifically evaluating its potential for enhancing our understanding of investigative interviewers’ approaches to questioning suspected child victims. The current research represented a critical step in this exploration, specifically focused on how forensic interview professionals questioned an AI child avatar about alleged maltreatment and perceived the avatar and interview process. Our results are directly relevant to the development of effective and engaging learning experiences (Felemban et al., 2017) for professional field interviewers who conduct interviews during maltreatment investigations.

Investigative interviewing is a critical skill for law enforcement, legal professionals, and child protective services (CPS) (Powell et al., 2010a, 2010b), all of whom at times question suspected child victims and witnesses to collect crucial details relevant to protection and prosecution. Interviewing children is particularly challenging, and interviewers need considerable expertise, knowledge, and practice to ask effective questions and elicit accurate, comprehensive, and reliable information (Benson & Powell, 2015; Lamb et al., 2018). Interviewers should follow “best practice” guidelines, which include primarily asking open-ended questions, minimizing the use of closed questions, and avoiding suggestive and leading questions (Lamb et al., 2007; Lyon, 2014). Field interviewers, however, have difficulty following these recommendations, and best practices are not always employed in interviews with suspected child victims (Lamb et al., 2018; Powell et al., 2010a, 2010b).

To maintain best practice approaches, interviewers need training. Traditionally, training in many countries has often been delivered via one-time half or full day evidence-based workshops, typically comprising lectures, slides, and videos. In the US, however, many forensic interviewers attend more intensive courses, such as those provided by the National Children’s Advocacy Center and the American Professional Society on the Abuse of Children, which include 30–40 hours of practice. Workshops are an efficient and cost-effective way of exposing large numbers of attendees to samples of questions that they should ask or avoid. Yet, such short training courses also have disadvantages. They usually offer only limited hands-on practice (Faller, 2014), primarily promote passive learning without active engagement, and they can only be attended by interviewers whose schedules fit the scheduled times and locations. Finally, during workshops, attendees are rarely given opportunities to practice, much less given feedback on their performance, and few follow-up sessions are provided to allow for ongoing learning (Akca et al., 2021; Lamb, 2016; Powell, 2008). Without intensive practice, tailored feedback, and repeated training over time, it is difficult to alter interviewers’ behavior in substantive ways (Lamb, 2016; Lamb et al., 2002).

Some innovative new studies have begun to test computer-based learning modalities as a way of evaluating interviewers’ skills (Brubacher et al., 2015; Casey & Powell, 2021; Haginoya et al., 2021; Kask et al., 2022; Pompedda et al., 2015; Powell et al., 2016), with the idea that these could ultimately be used to as training tools. Initial studies utilized a mechanized avatar, or an avatar overseen by an operator who answered interviewers’ questions. Different avatars were programmed to provide a range of responses, although because these were based on human operators, responses were somewhat limited, and the approaches were somewhat costly to program and operate. Nonetheless, hints emerged suggesting interviewers were reacting positively to the avatars as potential learning tools. Segal et al. (2024) exposed students to avatars describing child sexual abuse (CSA) scenarios. Students reported more intense negative emotions to the confirmed CSA case scenarios and relief to disconfirmed case scenarios. While the students’ facial expressions of emotion did not discriminate between the two scenarios as well, both the intensity of their emotional responses and the avatars’ perceived realism were reportedly key to their feelings. However, in this study, participants were only exposed to avatars in a 2D setting and they were not interviewing the avatars. Krause et al. (2024) found that integrating virtual reality (VR) into a training on child sexual abuse identification for student teachers increased their use of open-ended questions and feelings of self-efficacy relative to those reported by student teachers who had not been exposed to VR training. In combination, these studies suggest that it may be possible to immerse adults in VR environments simulating child sexual abuse interviewing. A crucial follow up question concerns whether that immersion leads adults to behave in ways that parallel how they would in actual interviews, or in other words, to question suspected child sexual abuse victims in the VR setting as they typically would. The perceived realism of a virtual character, in terms of presence and visual fidelity, may be vital in an educational training context to ensure the transfer of learning (Krause et al., 2024; Salehi et al., 2024).

To begin to pursue this question, in 2021, we expanded VR in a novel and exciting way by creating a multimodal AI-driven dynamic child avatar victim of abuse (Baugerud et al., 2021). We used Large Language Models (LLM) based on comprehensive question-and-response data from more than 741 well-designed mock interviews and some real-life interviews with suspected child victims which allows the avatar to respond to a variety of queries with relevance and coherence (Baugerud et al., 2021; Hassan et al., 2022a; Salehi et al., 2022) to program the avatar’s potential responses. Research with other LLMs has found that human-computer language interactions are comparable to human-human language interactions in VR settings (Heyselaar et al., 2017), and show promising results in simulated human conversations (Hadi et al., 2023). Further, LLMs like GPT-4 are equipped with a broad understanding of language, allowing them to be highly flexible with semi-supervised training when this is done using massive diverse datasets, like the interviews employed here. Our AI avatar’s responses in an interview, therefore, are not pre-programmed and an operator is not required, but instead are fully dependent on the interviewer’s questions. When adjusted and fine-tuned to specific applications, LLMs can show a high degree of sensitivity and precision in scenarios, including in child maltreatment interviews (Min et al., 2023). During training, via the fine-tuning of the specific LLM model, the avatar has become increasingly specialized in responding to interviewing questions to shape learning. We aimed to improve the model’s responsiveness to typical questions in interviewing settings, focusing on augmenting its ability to handle the complex emotional and psychological aspects that are important in interactions. The fine-tuning was designed to not only increase adherence to best practice, but also to incorporate empathetic and contextually sensitive responses, mimicking human-like responsiveness and, in our case, more childlike conversations.

When developing the avatar, we also combined different multimedia systems such as image, audio, and text (Hassan et al., 2022b) to create flexible 2D and 3D VR versions, and a chatbot version (Hassan et al., 2022b; Salehi et al., 2022, 2024). The system can also be programmed to code questions and responses in real-time, potentially offering feedback on interviewers’ use of different forms of questions immediately after an interview is complete (Hassan et al., 2023; Røed et al., 2023). Ultimately, the AI based avatar system could provide an innovative, ethical, and efficient solution to some of the challenges in current efforts to train interviewers, specifically, the high time and labor costs and limited opportunity to provide feedback over time (Powell et al., 2022; Salehi et al., 2022). We have begun to test the utility of the AI avatar in different contexts with different populations.

In one set of studies, we had child protection workers and students interact with the AI avatar on different platforms (2D desktop, 3DVR, audio, and text chat). Both the professionals and students reported feeling positive about the immersive and realistic AI environment, with some reporting that they favored it over 2D, audio and text chat and that 3DVR created higher presence and flow for users (Hassan et al., 2023). A critical next step in this work, though, is an assessment of how interviewers actually question and feel about the avatar, and how the avatar responds. If the avatar is to be useful in training, interviewers need to perceive the experience as realistic and treat the interview as they would one in the real-world. The avatar, in response to the interviewers’ questions, should respond in ways that parallel how children respond, but also in ways that are modifiable for different future training.

First, with regard to how professional interviewers typically question suspected child victims, it is universally accepted that the best way of eliciting a detailed and accurate narrative from a child is to ask non-leading open-ended questions (questions that are designed to elicit an elaborate and accurate response without dictating what specific information is required (Benson & Powell, 2015; La Rooy et al., 2015; Lamb et al., 2007, 2018). Yet, the most common questions actually asked by interviewers in interviews with suspected child victims are directive (e.g., who, what, when, etc., short-answer) and closed-ended (e.g., yes/no) in format, followed by suggestive questions (Baugerud et al., 2020; Johnson et al., 2015; Powell et al., 2010a; Yi et al., 2016). Closed-ended and directive questions yield less elaborate and accurate responses than recall prompts, as children are more inclined to provide single-word or short answers, and concurrently increase the risk of errors and suggestibility (Lyon, 2014; Lyon & Henderson, 2021). Insofar as professional interviewers react to and question the suspected child avatar in the same way that they do actual victims, we would expect similar patterns to emerge in VR settings. The avatar, in response, should mimic a conversational pattern, given that its programming was based on mock and actual interviews. That is, regardless of the form of abuse alleged, children tend to provide shorter and less informative responses to closed-ended questions than recall questions (Lamb et al., 2018). The avatar, in this proof-of-concept analysis, should do the same.

Second, turning to the learning potential, or value, of immersion, prior work has argued that high-speed computer communication provides ideal models for educational, training, and skill development (Jagatheesaperumal et al., 2022, preprint). However, this argument needs to be accompanied by data on how users actually feel about their digital experiences, especially with sensitive topics, such as in interviews regarding allegations of abuse (Hassan et al., 2022b). Investigating how users behave, their Quality of Experience (QoE), and their perception of the avatar’s realism are all pivotal (Newman et al., 2022), because these are closely linked to users’ level of engagement, which, in turn, influences learning outcomes (Allcoat & von Mühlenen, 2018). Users’ perceptions can also affect their motivations and effort during an activity, which also affect the efficacy of learning (Girvan & Savage, 2019; Hassan et al., 2023; Shershneva et al., 2014). Finally, positive perceptions are important. They can heighten users’ sense of presence, rendering a virtual experience more immersive and realistic, which again can improve training (Hess et al., 2022). It is imperative, therefore, to include an in-depth evaluation of users’ perceptions and experiences with the AI child avatar when evaluating its potential utility as a future interview training tool (Hassan et al., 2023; Weber et al., 2021).

The purpose of the present study was to address these issues and explore how professionals with experience interacting with and interviewing child victims questioned a child avatar victim of sexual or physical abuse. Of interest were the types of questions interviewers asked of the child avatar, how the avatar responded to the questions, and how interviewers perceived and experienced the interview and avatar. Evaluating the questions asked by interviewers is crucial for ascertaining whether they behave in expected ways when in an AI immersive environment. Evaluating the avatar’s responses is crucial to enhancing the AI avatar’s design and functionality. That is, by understanding the avatar’s response patterns, developers can refine the AI model to ensure more realistic and nuanced interactions. Finally, assessing users’ perceptions can help identify useful modifications. All of this, in turn, aids in the creation of a more effective and immersive training experience for professionals, thereby enhancing their interviewing skills in real-life situations.

## Present Study

In this study, we examined professionals’ questions and perceptions during a mock VR forensic interview with a dynamic AI-based child avatar. We included two scenarios involving suspected abuse, one involving child physical abuse (CPA) and the other child sexual abuse (CSA), to assess the generalizability of the AI avatar and immersive VR. Even though at times disclosure patterns vary between child victims of CPA and CSA (Ahern et al., 2014; Azad & Leander, 2015; Eisen et al., 2021; Rush et al., 2014), we did not expect robust differences between interviewers’ questions or perceptions in the two abuse scenarios. We nonetheless tested for differences, under the assumption that such differences would be relevant to needed modifications and ultimately the development of training protocols for interviewers in the future. Regardless of abuse type, though, we expected interviewers to ask proportionally more closed-ended questions than recall questions. We expected relatively few but some leading questions, given that all the participants were professionals with prior experience and training in the field working with child maltreatment victims. We anticipated that the child avatar would be more productive in response to recall than closed-ended questions.

## Method

### Participants

Sixty-eight professionals (56 female, 12 male) served as participants. All were attending the 38th Annual San Diego International Conference on Child Maltreatment (https://www.chadwickcenter.org/) and hence were interested in topics related to the prevention of, identification of, and interventions following exposure to child maltreatment. Participants’ ages ranged considerably: (9% 18–29 years; 31% 30–39 years; 21% 40–49 years; 31% 50–59 years; and 9% 60 years and older). Self-reported occupations included interviewer (35%: forensic interviewer, law enforcement, police), medical professional (18%: medical student, resident, physician, nurse practitioner), social worker (16%), academic/researcher (9%), psychologist (10%), or other/unclear (11%; e.g., attorney, manager, service professional). Finally, 19% of the participants reported no formal experience interviewing children, 13% reported less than a year, 22% reported between 1–5 years, 10% reported between 5–10 years, and 35% reported more than 10 years.

Of the 68 participants, 35 questioned a suspected child avatar victim of sexual abuse, and 33 questioned a suspected child victim of physical abuse. One participant completed the interview via 2D, and 67 completed the interview via 3D VR.

### Materials

#### Child Avatar VR System

The Child Avatar Interview Program was created using the unity^1^ game engine to be used in VR with Oculus Quest 2^2^. The visual components were crafted with customization capabilities using the open-source Unity Multipurpose Avatar (UMA) project, supplemented with the Salsa Suit asset to generate eye, head, and lip movements synchronized with voices. The auditory components leveraged IBM Watson services for speech-to-text (STT) and text-to-speech (TTS) synthesis, adjusted to replicate child-like sounds. The language model is the core of the backend system, which utilized a fine-tuned GPT-3 (Brown et al., 2020), trained using mock interviews (*n* = 741) sourced from a forensic interview training program by the Centre for Investigative Interviewing at Griffith University (Powell et al., 2016, 2022). The model trained across two distinct scenarios, one sexual abuse (CSA) and one physical abuse (CPA), based on 10 mock forensic interviews scenarios based on anonymized paraphrased real-life interviews with suspected child victims ages six to eight years, in order to generate dynamic and contextually relevant answers to interviewers’ questions.

#### Demographic and User Experience Questionnaire

We collected demographic information (age, occupation, gender, years of experience interviewing children) and information about participants’ experiences in the interview and interacting with the avatar. Age was reported as one of the following: 18–29 years; 30–39 years 40–49 years; 50–59 years; and 60 years and older. Five-point Likert questions focused on participants’ experiences with the avatar in the interview. Three asked how realistic the child avatar was in appearance, speech, and expressions (response options ranged from 1 = *very unrealistic* to 5 = *very realistic*). Two asked whether the child avatar’s responses seemed normal or were random (response options ranged from 1 = *strongly disagree* to 5 = *strongly agree*), and one asked how comfortable participants were interviewing the child avatar (1 = *very uncomfortable* to 5 = *very comfortable*). Three open-ended questions were also asked about participants’ experiences: how they felt about interviewing a child avatar, what caught their attention the most, and how the platform could be improved.

### Procedures

All procedures were approved by the Norwegian Agency for Shared Services in Education and Research (SIKT) under project number 614272 and Oslo Metropolitan University, the institution that oversaw data collection. Participation was voluntary and confidential, and none of the data was linked to any personal information. To complete the study, participants followed separate links for the consent form, interview, and demographic and user experience questions, and no identifiers connected the three links. Finally, no personal data were retained or stored following data collection.

#### Recruitment

With support of the Conference Organizing Committee, recruitment was done during the conference. Announcements were made in several conference sessions and in the opening meeting about the Child Avatar Interview Training Program and our study, directing interested attendees to the exhibition hall to learn more. At a booth in the hall, attendees could ask questions. Those who wished to take part were directed to a separate study room where two VR systems (monitors, keyboards, headsets) were located on opposite sides of the room, each with a researcher who introduced participants to the equipment, reviewed the consent, and coordinated data collection.

#### Interview Sessions

Across the four days of the conference, 76 individuals took part. Errors arose in the VR equipment or program that stopped data collection for eight individuals, leading to our final sample of *N* = 68. The participants were told that we were developing an online VR-child interviewing paradigm and were interested in their experiences and thoughts regarding the program. The participants were given the link to the consent form to review.

Next, participants were given a link to the Child Avatar Interview program and were told they had 5–10 minutes to find out what, if anything, had happened to a child. Participants were informed of the specific form of alleged abuse, as follows: Participants in the CSA condition (*n* = 35) were told that their job was to interview a child to find out about possible exposure to sexual abuse. Participants in the CPA condition (*n* = 33) received the same instructions, with the reference changed to physical abuse. They were told that the session did not represent a complete lengthy investigative interview; rather, they could engage in a brief introductory phase and then focus on substantive questioning to ascertain what, if anything, happened. Participants were not instructed to build rapport, although a few elected to do so. In addition, a few participants paused mid-way and asked the researcher questions or made comments (e.g., “Should I keep going?” “Do all children respond the same?”). The researcher answered briefly that the participant just should go on and directed the participant back to the interview. Once participants indicated they had finished, they were given the link to the post-interview demographic and experience questionnaire, and when they were finished, they were thanked, and their questions were answered.

### Coding

Questions were coded into one of four mutually exclusive categories following schemes used in prior studies of interviewer questioning behavior in forensic settings (e.g., Ahern et al., 2018; Evans et al., 2014). Categories included *free recall-based questions* (e.g., Invitations, e.g., “Tell me what happened,” and *cued recall-based questions* (e.g., “What happened then”, Wh prompts, such as “Where were you when her dad came over?”); *option-posing* (yes/no and forced choice, e.g., “Is there something else that happened?), *leading* (tag questions, statement questions; e.g., “I wonder what made his hands all wet?”), and *do you know/remember* questions (DYK/DYR) (e.g., “Do you want to start from the beginning and tell me what happened?” “Do you know what you). Reliability was established on a separate dataset of interviewer and attorney questioning of alleged child victims of CSA (*N* = 6653 questions, Cohen’s *K* = .85). Two coders reviewed the questions and each coded half of the interviews.

Each answer provided by the avatar was coded for productivity, calculated as the number of words produced (a small number of nonsensical words or phrases was removed).

## Results

First, we evaluated participants’ approaches to questioning the child avatar in a VR context. We present descriptive data and then analyses of the proportion of each question category. We compared these proportions between participants who questioned the suspected CSA and CPA child avatar victim. Second, we examined the effects of questions on the avatar’s productivity, examining whether the total number of words varied as a function of the question category and between the two types of victims. Third, we explored participants’ perceptions of the child avatar, including whether their perceptions varied as a function of abuse type. Again, we did not expect differences between participants who interacted with the CSA and CPA child avatars, but we explored whether any existed.

### Interviewer Questions

Overall, participants asked 1358 questions of the avatar, with participants asking on average 20 questions (*M* = 19.97, *SD* = 11.61; range 4 to 54). The most common category was recall based, followed by option-posing and DYK/DYR. Leading questions were relatively infrequent, although the number was nonetheless sizable (Table 1).

**Table 1.** Question Type Frequencies (Total 1358 of Questions).

Question type	Sexual abuse	Physical abuse	Overall
Option-posing	15% (*n* = 67)	24% (*n* = 219)	21% (*n* = 286)
Leading	5% (*n* = 21)	8% (*n* = 73)	7% (*n* = 94)
Open-ended	62% (*n* = 276)	58% (*n* = 531)	60% (*n* = 808)
Open-ended (no invitations)	52% (*n* = 230)	51% (*n* = 465)	51% (*n* = 696)
Invitations	10% (*n* = 46)	7% (*n* = 66)	8% (*n* = 112)
Do you know/remember	18% (*n* = 80)	10% (*n* = 90)	13% (*n* = 170)

When we compared the proportion of each question category between participants who questioned the suspected CSA victim versus the suspected CPA victim, several differences emerged, χ2 (3) = 32.57, *p* < .001, Cramer’s *V* = .16. Participants asked proportionally similar numbers of recall-based questions of the two types of victims. However, participants questioning the suspected CSA victim asked proportionally more DYK/DYR questions (Table 1), whereas participants questioning the suspected CPA victim asked proportionally more option-posing and leading questions.

### Child Avatar Responses

We next analyzed the child avatar’s responsiveness, focusing on whether the length of the avatar’s answers varied across question types via a 2 (Abuse type: CSA vs. CPA) x 4 (Question category: recall-based, option-posing, leading, DYK/DYR) mixed model ANOVA predicting the child avatar’s productivity. Abuse type varied between subjects, and question category varied within subjects. Both main effects (abuse type, *F* (1, 1350) = 129.15, *p* < .001; question type, *F* (3, 1350) = 29.29, *p* < .001) were significant. Regarding the abuse type effect, as is evident in Table 2, the suspected CSA child avatar was consistently more productive, regardless of the question category, than the suspected CPA child avatar. The question category effect, examined via Bonferroni-corrected follow-up comparisons, revealed that child avatars were more productive to recall-based than option-posing [*t* (1092) = 11.49, *p* < .001, *d* = 0.79] and leading [*t* (900) = 6.72, *p* < .001, *d* = 0.73] questions. No differences in productivity emerged between DYK/DYR and the other question types: recall-based [*t* (976) = 1.94, *p* = .05, *d* = 0.16], option-posing (*t* (454) = 6.77, *p* < .001, *d* = 0.66], and leading [*t* (262) = 4.45, *p* < .001, *d* = 0.57].

**Table 2.** Perceptions Ratings Descriptives (5-Point Scale Ratings).

Question	Sexual abuse (*n* = 35)	Physical abuse (*n* = 33)	Overall (*N* = 68)
How realistic was avatar talking?	3.54(0.85)	3.33(1.02)	3.44(0.94)
How realistic was avatar appearance?	3.17(1.12)	3.64(0.90)	3.40(1.04)
How realistic was avatar expression?	3.11(0.96)	3.00(0.90)	3.06(0.93)
Your comfort engaging the avatar	3.46(0.98)	3.73(0.84)	3.59(0.92)
The avatar responses seemed normal	3.94(0.80)	3.82(0.88)	3.88(0.84)
The avatar responses seemed random	2.26(0.92)	2.06(0.70)	2.16(0.82)
The avatar’s responses seemed delayed	3.94(0.73)	4.12(0.74)	4.03(0.73)

*Note.* Values in cells represent mean scores, values in parentheses in cells represent standard deviations.

### Perceptions of the Child Avatar

Our final analyses focused on the participants’ perceptions of the VR program and the child avatar. Given the goal of the program is to create a method of training interviewers on best practices, it is crucial to know professionals’ thoughts on the program as it is developed. We focused on how realistic professionals considered the avatar and the avatar’s responses to be, how comfortable they were with the program, and what aspects need to be improved.

First, we considered participants’ ratings of how realistic the avatar seemed in her speech, appearance, and expressions. Participants rated all three characteristics as somewhat realistic (Table 2). When the three ratings were entered into separate 2 (Abuse type) ANOVAs, none of the models was significant, *Fs* (1,66) < 3.53, *ps* > .065. Thus, participants did not see the avatar differently depending on whether she was alleging CSA or CPA. Next, we evaluated participants’ ratings of their agreement with statements regarding the avatar’s responses seeming normal, random, and delayed. Participants were fairly consistent in their views that the avatar was answering in a normal and not random manner, but participants did indicate that the answers seemed delayed. Abuse type ANOVAs failed to uncover differences between conditions in participants’ agreement ratings, *Fs* (1,66) < 1.01, *p* > .319, meaning that participants viewed the response tendencies similar between the two victim types.

Second, we evaluated what participants thought of the avatar and their experiences. Participants seemed to feel fairly comfortable with the system, as suggested by their mean comfort scores (Table 2). An abuse type ANOVA revealed that participants’ comfort did not differ between scenario conditions, *F* (1,66) = 1.48, *p* = .22. Similarly, when answering an open-ended question about how they felt about their experience, a majority of participants (*n* = 41) wrote positive feelings (e.g., “good,” “exciting,” “cool,” and “love it”). A smaller number (*n* = 23) wrote ambiguous or positive and negative feelings (e.g., “interesting,” “potentially useful,” “good and bad”). Finally, four participants provided only negative comments concerning their experience (“slow,” “bad”). Responses to the two other open-ended questions, namely what was most noticeable and what could be improved, were also largely positive with some important caveats. The largest number of participants (*n* = 21) mentioned that the avatar’s behavior was realistic, including in her language, how she phrased her responses, or her fidgeting and eye contact. A few participants mentioned that the pauses when answering were realistic, but a larger number of participants (*n* = 17) mentioned the delay between the participants’ questions and avatar’s responses as an area in need of improvement. The third most common theme, noted by 12 participants, was that the avatar did not physically look like a 7-year-old, but instead appeared to be much older.

## Discussion

The overarching purpose of the present study was to evaluate the potential utility of a dynamic AI based child avatar system to understand and ultimately improve investigative interviews with suspected child victims of maltreatment. Our findings indicate that the fine-tuned LLM successfully mirrored key aspects of children’s responses observed in real interviews, demonstrating the potential of LLMs in effectively supporting sensitive and nuanced interviewer-avatar interactions in child abuse and maltreatment cases.

### Interviewer Questions

Our first goal focused on how interviewers engaged with the child avatar, that is, what types of questions they asked and whether their behavior paralleled what one might expect in actual forensic interviews. At simply the level of overall numbers, percentages of each question type suggest that participants used a wide array of prompts to elicit abuse disclosures from the child avatar. This is similar to what is observed when interviewers conduct actual forensic interviews, especially among interviewers trained in best practice approaches (Ahern et al., 2018; Cederborg et al., 2013). What was somewhat surprising was the high numbers of recall-based questions, which comprised 60% of all substantive questions. Thus, participants attempted to elicit more details from the child avatar, as would be expected in forensic interviews. Studies of the National Institute of Child Health and Human Development (NICHD) forensic interviewing protocol suggest that over 50% of interviewers’ questions, post-training, are open-ended in format (Lamb et al., 2018).

Because the 60% we observed combines recall invitations and directives (WH- questions) (e.g., Lamb et al., 2009), and recall invitations are widely considered ideal prompts to use when first attempting to elicit disclosures from children (Saywitz et al., 2017), we reviewed our open-ended questions and identified only those classified as invitations (Lamb et al., 2007). Invitations are broad requests for children to describe everything they remember and follow-up “tell me more prompts” that asked for additional details about topics children just mentioned. Overall, 14% of the open-ended questions, or 8% of the total questions, were invitations. These percentages are consistent with those reported in prior work (e.g., Baugerud et al., 2023; Orbach et al., 2000), which found that about 10% of the prompts asked by forensic interviewers not trained to use the NICHD Protocol were invitations. Our participants, therefore, performed much like other professionals, which perhaps is a function of both where they were recruited and the nature of the task itself. All participants were attending a conference devoted to topics concerning identification and intervention of child maltreatment. Participants were immersed in reminders about best practices or had experience and training on these approaches. In addition, the context may have lent itself to high self-monitoring. The avatar is designed to be realistic. Thus, participants may not have felt the same level of pressure to elicit a disclosure from the avatar that they typically feel in real interviews, which enabled them to rely heavily on recall and open-ended prompts to gather details. The child avatar also was designed to elaborate to open-ended prompts, and participants were probably behaving in a way that reflected the quality and quantity of their prior training.

While we did not expect differences in the types of questions asked of the CSA and CPA victims, a few emerged that are worth considering in future research and training. Many cases of CSA involve contrasting reports from child and adult participants, and interviewers may attempt to be cautious when questioning suspected CSA victims so as not to lead them into eliciting false information. Here, that caution could be reflected in DYK/DYR questions, which interviewers often mistake as a form of open-ended prompt (Evans et al., 2014, 2017), because they implicitly request narrative details (e.g., “Could you tell me about how that happened?” “Do you remember what he did next?”) while explicitly requesting a ‘yes’ or ‘no’ answer. Physical abuse cases, in contrast, are more likely to contain other forms of evidence, such as marks or bruises (Rush et al., 2014), and concerns about children’s errors might not be as salient. Thus, interviewers may not need to rely as heavily on a child’s claims or may feel freer to ask option-posing questions about targeted details. Because we included only one scenario of each type, we are limited in what we can conclude. Nonetheless, variations in interviewers’ questioning strategies as a function of the type of abuse is an important direction for future research, both in terms of testing the utility and generalizability of an avatar-based interviewing training program, but also in terms of promoting interviewing best practices broadly.

### Child Avatar Responses

The second goal of our study concerned how the child avatar responded. Results revealed four important sets of findings. For one, the avatar was indeed responding with more detail to questions designed to elicit further elaboration, namely recall-based questions, as intended and as children typically do (Cederborg et al., 2000; Gagnon & Cyr, 2017; Lyon, 2014). Thus, at the level of programming, the fine-tuned GPT-3 (Brown et al., 2020), created using mock forensic interviews, was effective. Second, insofar as interviewers can be trained to use recall-based prompts, having an avatar that responds in kind by providing lengthy narratives could be a valuable form of positive reinforcement for interviewers.

Third, findings also point to the need for more nuanced training of the avatar, specifically in relation to DYK/DYR questions. Indirect speech act questions, particularly those that vary in explicit and implicit requests (e.g., Can you tell me about the time when that happened?) can be confusing and lead to varying answers in children. The avatar, though, was nearly as productive to the DYK/DYR prompts as the recall-based prompts, suggesting that the avatars were not discriminating between recall-based and DYK/DYR questions when responding. Interviewers similarly may have difficulty distinguishing these (Lyon & Henderson, 2021). Young children, however, regularly have difficulty with DYK/DYR questions, leading them to provide abbreviated responses when they answer explicit prompts (e.g., Evans et al., 2014, 2017). Further programming of the avatar could be used to alter interviewers’ use of these questions. For instance, the avatar could be programmed to provide elaborated answers only to invitations and open-ended questions and respond minimally or even in minimally comprehensible ways to the most problematic questions (Brubacher et al., 2015; Casey & Powell, 2021). This would reinforce the utility of recall-based questions for interviewers and align with their expectations of how responsive a victim will be to these types of questions, potentially leading to alterations in questioning approaches that have, until now, been particularly difficult to induce (Hadi et al., 2023).

A fourth issue that emerged in our examination of the avatar’s responses, and one that is worth considering in future research, concerned the productivity differences in the avatar describing sexual abuse versus physical abuse. The avatar describing sexual abuse provided substantially more details, regardless of the format of the question, compared to the avatar describing physical abuse. Such could be a function again of how the AI avatar was trained, specifically with mock interviews which could have included more details in sexual than physical abuse claims. However, it may also be of interest to unpack how interviewers’ approach legal questioning of suspected child victims of sexual versus physical abuse, as well as how victims’ productivity and content vary between the two. In doing so, we would move beyond just collapsing across abuse types in analyses of forensic interviewers (Baugerud et al., 2020; Hershkowitz et al., 2006) and instead be able to understand variations that are important for children’s disclosures, report completeness, and accuracy.

### Interviewers’ Perceptions

The final goal of our study concerned user experience, that is, how the interviewers felt while questioning the child avatar and what they thought of the avatar’s behavior. We focused on the participants’ perceptions of the VR program and child avatar itself. Participants’ evaluations of the avatar’s realism in terms of speech, appearance, and expressions were quite positive, and most felt fairly comfortable engaging with the avatar. Given the potential interplay between learner engagement, motivation, and avatar presentation, and how these factors may influence perceived usefulness, it is crucial to investigate these connections further. Such an understanding is vital for mapping out users’ intentions to use the avatar as a tool for interview training. Furthermore, our results showed that participants did not see the avatar differently depending on whether she was alleging CSA or CPA. Participants also felt that the avatar answered normally, and in a not random but somewhat delayed manner.

However, though most of the participants reported positive feelings in relation to this immersive experience, some also reported mixed or negative attitudes towards the avatar. Some participants found the pauses in the avatar’s response time realistic, yet others felt that this delay between the interviewers’ questions and the avatar’s responses is an area in need of improvement. This highlights the importance of ongoing efforts to improve the quality and realism of responses by leveraging both the advances made in large language models (LLMs) as well as in conjunction with the use of more realistic-looking avatars (Hassan et al., 2023). Finally, it will also be important to align the visual appearance of avatars with their stated age. If users expect their interaction with a 6-year-old avatar to resemble their interaction with a real child of the same age, the avatar must accurately reflect a 6-year-old in appearance and not appear significantly older. Overall, despite some needed improvements, which are inevitable as new programs are being developed, participants’ perceptions of the program and the avatar are all quite positive, showing they valued and felt comfortable with the VR training.

### Limitations

The novelty and potential value of our investigation should be evaluated in conjunction with limitations, which also affect the conclusions, generalizability, and next steps in this line of inquiry. One is that the participant pool, drawn exclusively from attendees of the San Diego International Conference on Child Maltreatment, may not represent the broader demographic of professionals in the field, potentially limiting generalizability. Additionally, the AI avatar, despite its sophistication, might not entirely encapsulate the complex behavioral nuances of child victims, and it needs to shape learning which could influence the depth of training and its applicability in real-world settings. We also focused only on two specific abuse scenarios (CSA and CPA), an important first step to generalization but potentially limiting the broader applicability of our findings. In addition, it should be noted that the simulated avatar-based interviews in the present study lasted, on average, about 10 minutes, whereas real-life interviews often last for at least 1 hour and frequently extend with breaks in between. There is also a need to explore various training scenarios in which avatars exhibit reluctance to disclose abuse. In real-life investigative interviews, children often only partially disclose or do not disclose at all, highlighting the importance of conducting training with avatars simulating these scenarios. Such training may particularly benefit the incremental learning of interview skills, but it may pose challenges for novice interviewers, as handling an avatar that does not disclose can potentially affect their motivation for training. Furthermore, the study’s reliance on self-reported data might introduce subjective biases, affecting the interpretation of the avatar’s effectiveness. Lastly, technical limitations inherent in the VR system and AI model could have impacted the avatar’s response dynamics and overall training experience quality. These factors should be considered when interpreting the study’s findings and serve as possible focal points for further research. Nevertheless, our findings offer an important first step towards implementing an effective and immersive forensic interviewing training program.

### Conclusions

We evaluated interviewer conduct in interactions with a child avatar, focusing on the nature of interviewer questions, the authenticity and user experience, and the avatar’s ability to exhibit human-like responses. Our findings indicate that interactions with the avatar may mirror real-world investigative interview practices. Participants perceived the avatar as a valuable tool for honing forensic interview skills. These results underscore the potential of AI-driven avatars as a novel, standardized, and scalable approach to interview training, offering a significant advancement and in addition to traditional methods. Further research should focus on refining the avatar’s response mechanisms to various question types and test its adaptability across different scenarios and with diverse user groups, thus paving the way for more effective and versatile interview training techniques.

## Notes

1.
https://unity.com/

2.
https://www.meta.com/quest/
